# Analysis of the Intrinsic Self-Organising Properties of Mesenchymal Stromal Cells in Three-Dimensional Co-Culture Models with Endothelial Cells

**DOI:** 10.3390/bioengineering5040092

**Published:** 2018-10-26

**Authors:** Julia Marshall, Amanda Barnes, Paul Genever

**Affiliations:** Department of Biology, University of York, York YO10 5DD, UK; julia.marshall@gmail.com (J.M.); amanda.barnes@york.ac.uk (A.B.)

**Keywords:** 3D in vitro models, MSCs, endothelial cells, osteoblasts, chondrocytes, self-organisation, vascularization, regenerative medicine

## Abstract

Mesenchymal stem/stromal cells (MSCs) are typically characterised by their ability to differentiate into skeletal (osteogenic, chondrogenic and adipogenic) lineages. MSCs also appear to have additional non-stem cell functions in coordinating tissue morphogenesis and organising vascular networks through interactions with endothelial cells (ECs). However, suitable experimental models to examine these apparently unique MSC properties are lacking. Following previous work, we have developed our 3D in vitro co-culture models to enable us to track cellular self-organisation events in heterotypic cell spheroids combining ECs, MSCs and their differentiated progeny. In these systems, MSCs, but not related fibroblastic cell types, promote the assembly of ECs into interconnected networks through intrinsic mechanisms, dependent on the relative abundance of MSC and EC numbers. Perturbation of endogenous platelet-derived growth factor (PDGF) signalling significantly increased EC network length, width and branching. When MSCs were pre-differentiated towards an osteogenic or chondrogenic lineage and co-cultured as mixed 3D spheroids, they segregated into polarised osseous and chondral regions. In the presence of ECs, the pre-differentiated MSCs redistributed to form a central mixed cell core with an outer osseous layer. Our findings demonstrate the intrinsic self-organising properties of MSCs, which may broaden their use in regenerative medicine and advance current approaches.

## 1. Introduction

Traditional two-dimensional (2D) cell culture techniques have provided valuable insights into fundamental aspects of cell function. However, it is clear that plastic-adherent culture environments cannot fully recreate the complexity of 3D tissues in vivo. Sophisticated 3D co-culture models can provide tractable systems to examine the mechanisms controlling the organisation and interaction of heterotypic cells, and may be developed for more effective forms of cell-based therapy. The use of mesenchymal stem/stromal cells (MSCs) in therapy has primarily been targeted towards regenerative applications, largely through their ability to differentiate into bone and cartilage tissues [1,2,3]. MSCs also appear to possess many, apparently unique, additional properties that enable them to modify the function of diverse cell types through paracrine signalling activity and/or cell–cell interactions, opening up new treatment options [4,5,6]. Most therapies however rely on the ex vivo expansion of MSCs in 2D culture and there are uncertainties regarding the true nature of the in vitro MSC compared to its in vivo counterpart [7]. Convincing evidence suggests that bone marrow MSCs occupy a perivascular location, closely associated with endothelial cells (ECs) [8,9,10,11,12,13], and that MSCs are able to influence vascular remodelling [8] and direct bone marrow vascularisation to couple with osteogenesis [14,15]. Similarly, long bone development through endochondral ossification and osteochondral tissue repair both involve a coordinated interplay between ECs and mesenchymal cells, ultimately to generate vascularised bone tissue and avascular cartilage [16]. It is important therefore that we have appropriate tools to explore the relationship between MSCs and ECs to expand basic understanding of MSC biology and develop therapeutic applications, for example by engineering vascularised replacement tissues [17], and high-throughput screening platforms [18].

We and others have previously developed 3D in vitro models to examine interactions between MSCs with ECs [19,20]. These studies demonstrated that ECs promoted MSC osteogenesis most likely through paracrine signalling activity. We also observed that MSC:EC co-cultures in an unsupported 3D spheroid, self-organised to form elaborate endothelial lattices in the absence of exogenous cues [19]. Here, we have explored the self-organisation involved in MSC:EC and determined how the presence of osteogenic and chondrogenic elements, differentiated from MSCs, influence this process.

## 2. Materials and Methods 

### 2.1. Cell Culture

Human bone marrow MSCs were extracted from bone samples obtained during joint replacement surgery following informed consent as previously described [21]; donors were fully anonymised. All MSCs tested positive for CD29, CD44, CD73, CD90 and CD105, and negative for the haematopoietic markers CD34 and CD45 and the endothelial marker CD31 by flow cytometry (not shown). MSCs were cultured in Dulbecco’s Modified Eagle Medium (DMEM) (Gibco, Waltham, MA, USA) with 15% batch tested foetal bovine serum (FBS) (Biosera, Boussens, France) and 100 U/mL Penicillin/100 μg/mL Streptomycin (P/S) (Life Technologies, Waltham, MA, USA). MSCs were used in experiments up to passage 5. ECs (human umbilical vein endothelial cells) were obtained commercially (PromoCell, Heidelberg, Germany) and maintained in Endothelial Cell Growth Medium (ECGM) (PromoCell) with the provided supplement mix, 10% FBS and P/S. ECs were CD31-positive (not shown) and used between passages 4 and 6. Human dermal fibroblasts (HDFs) were purchased from Cascade Biologics (Life Technologies) and cultured in DMEM containing 15% FBS and P/S. HDFs in all experiments were used between passage 7 and 10. All cells were cultured at 37 °C in 5% CO_2_/95% air humidified atmosphere. On reaching confluency were passaged using 0.05% trypsin, 0.02% EDTA (Life Technologies).

### 2.2. MSC:EC Spheroid Formation and Fluorescent Labelling

Co-cultured spheroids were created by combining MSCs and ECs at different cell ratios to generate MSC:EC spheroids; MSC:EC percent ratios examined were 20:80, 35:65, 50:50, 65:35 and 80:20, all spheroids had a total of 30,000 cells, therefore the 80:20 MSC:EC spheroid contained 24,000 MSCs and 6000 ECs. To enable fluorescent tracking, the MSC population was stained using CellTracker™ green (Life Technologies) whilst ECs were labelled with CellTracker™ red (Life Technologies). Control MSC-only spheroids were comprised of a 50:50 mix of green-labelled MSCs and red-labelled MSCs. The different cell types were trypsinised and counted before being washed with phosphate buffered saline (PBS), they were then incubated in the appropriate CellTracker™ green or CellTracker™ red for 45 min before the remaining CellTracker™ was neutralised and removed. The cells were then combined at the different ratios in a 50:50 mix of DMEM:ECGM medium containing 0.25% (*w*/*v*) methylcellulose (Sigma Aldrich, St. Louise, MO, USA) to support 3D co-cultured spheroid formation and cell viability, as previously described [19]. The mixed cells were then seeded into non-adherent U-bottomed 96-well plates (Fisher Scientific, Waltham, MA, USA) and incubated at 37 °C to generate spheroids in 100 μL medium containing 30,000 cells per spheroid per well. Medium was changed twice a week by split feeding to avoid disturbing the spheroid. HDF:EC spheroids were generated as above, replacing MSCs with HDFs.

### 2.3. Inhibitor Treatment of MSC:EC Spheroids

To help identify signalling pathways involved in MSC:EC spheroid organisation, the 3D cultures were exposed to a panel of different inhibitors. These inhibitors targeted integrin-linked kinase (ILKi, 2 μM, 407331, Millipore, Darmstadt, Germany), platelet-derived growth factor receptor (PDGFRi, 3 μM, sc 205794, Santa Cruz, Dallas, TX, USA), fibroblast growth factor receptor (FGFRi, 4 nM, 3044, Sigma Aldrich), epidermal growth factor receptor (EGFRi, 100 nM, 1037, Tocris, Abingdon, UK) and Notch (Dibenzazepine, DBZ, 3 μM, 4489, Tocris). Further information on inhibitor specificity and selection can be found in Table S1. The inhibitors were added to cell suspensions post-labelling prior to pipetting into U-bottomed 96 well plate to induce spheroid formation. Spheroids were then maintained in 50:50 DMEM:ECGM, with or without the various inhibitors for up to 7 days. Inhibitors were replenished at each medium change. 

### 2.4. Generation of Mixed Cell Spheroids Containing ECs and MSCs Pre-Differentiated Into Osteogenic and Chondrogenic Lineages

Osteogenic differentiation of MSCs was induced by culturing the cells in osteogenic medium containing 5 mM β-glycerophosphate, 50 μg/mL ascorbic acid phosphate and 10 nM dexamethasone (all Sigma-Aldrich) in DMEM supplemented with 15% FBS and P/S, for 7 days in 2D culture. Chondrogenic differentiation of MSCs was induced by culturing the cells in chondrogenic medium consisting of 50 µg/mL L-ascorbic acid, 100 nM dexamethasone, 40 µg/mL L-proline (Sigma-Aldrich), 1% ITS + Universal culture supplement premix (VWR International, Randor, PA, USA) and 10 ng/mL TGF-β1 (PeproTech, Rocky Hill, NJ, USA) in DMEM, supplemented with P/S for 7 days in 2D culture. It should be noted that chondrogenic differentiation is more effective in high cell density 3D pellet culture, however it was difficult to disaggregate the 3D chondrogenic pellets into single cell suspensions, prior to recombining as spheroids, without adversely affecting viability.

Pre-differentiated MSCs were then used to create multicellular 3D spheroids. Osteogenic MSCs were labelled with CellTracker™ red, chondrogenic MSCs were labelled with CellTracker™ green and ECs were labelled with CellTracker™ blue. These three different cell types were then pooled in equal combinations to make osteo and/or chondral spheroids with and without ECs containing a total of 30,000 cells per spheroid. Cell combinations were: (1) Osteogenic MSCs with chondrogenic MSCs (OC); (2) Osteogenic MSCs with ECs (OE); (3) Chondrogenic MSCs with ECs (CE) and; (4) Osteogenic MSCs with chondrogenic MSCs and ECs (OEC).

### 2.5. Image Analysis

Whole spheroids were imaged using multiphoton confocal microscopy (Zeiss LSM 780 multiphoton invert microscope, Zeiss, Oberkochen, Germany). Images were captured at the indicated time-points within the U-bottomed 96 well plate. Parallel spheroid samples were snap frozen in liquid nitrogen and embedded in optimal cutting tissue (OCT) medium (Tissue-Tek, Sakura Finetek, Tokyo, Japan). Spheroids were cryosectioned at 10 μm thickness (Bright OFT5000 cryostat, Bright Instruments, Luton, UK) and imaged using multiphoton confocal microscopy (Zeiss LSM 780) to observe internal cellular organisation. Microscopy was used in combination with Zen imaging software (Zeiss), Volocity software (Perkin Elmer, Waltham, MA, USA) and Image J (McMaster Biophotonics Facility) to process and analyse images. The plot profiler extension on Image J was used to analyse spheroid cross-sections. Each spheroid section had a minimum of three different z-axes drawn across the width of the spheroid along a centre-line. The plot profiler was then used to calculate the amount of either green or red fluorescence at a specific point on each axis and averaged to determine green-labelled and red-labelled cell grouping. The freehand extension on Image J software was used to quantify the length and width of endothelial cell networks within spheroid sections. The number of branches was calculated manually. 

### 2.6. Statistical Analysis

Data show mean ± standard deviation unless otherwise stated. Experiments were performed at least in triplicate using three separate, unpooled biological donors in three independent experiments. All data were analysed using PRISM software (GraphPad, La Jolla, CA, USA); one-way and two-way ANOVA was performed on appropriate data sets with Tukey post-test or Bonferroni post-test. *p* values <0.05 were considered statistically significant. * *p* < 0.05, ** *p* < 0.01, *** *p* < 0.001, **** *p* < 0.0001.

## 3. Results

### 3.1. Development of MSC:EC Spheroids

**Figure 1:**
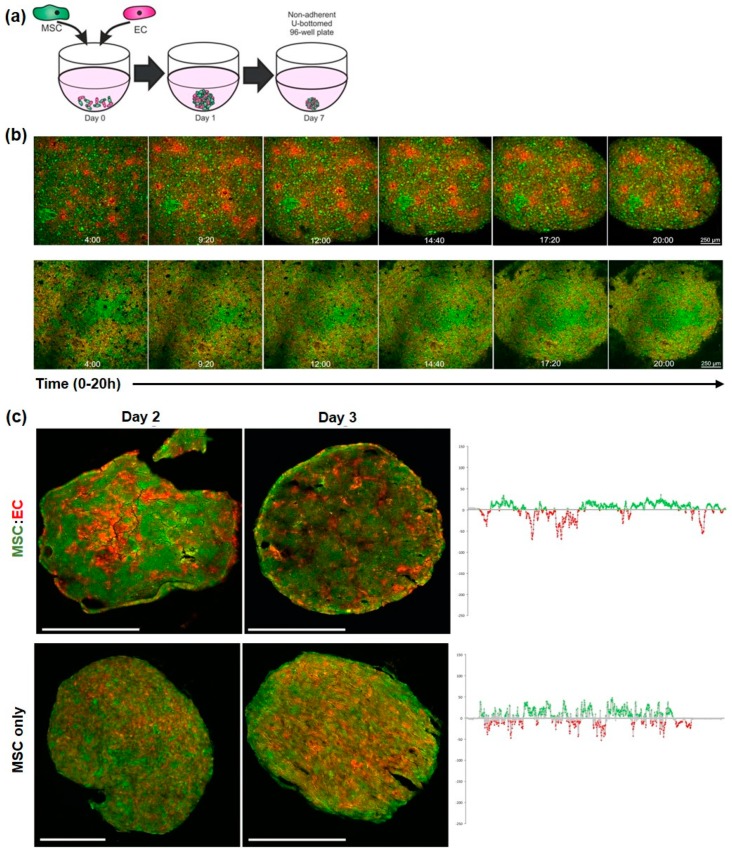
Fluorescent imaging of MSC-ECs during spheroid formation. (**a**) Schematic showing technique for combining MSCs and ECs to form co-cultured spheroids containing 30,000 cells in total per well of the non-adherent U-bottomed 96-well plate. (**b**) Top: Time-lapse images of MSC-EC (50:50) spheroid formation; MSCs labelled green, ECs labelled red; Bottom: MSC-only control containing 50:50 MSC (green):MSC (red). Images were captured every 20 min from 4 h to 20 h, sample time points shown. (**c**) Higher magnification images of MSC:EC (top) and MSC-only (bottom) spheroid sections at days 2 and 3 of culture, all scale bars = 200 μm. Graphs on the right quantify green-red cell distribution across the centre line of sections from day 3 spheroids using the plot profiler extension on Image J. The data show the distribution of green-labelled cells as positive values above the x-axis and red-labelled cells as negative values below the x-axis.

Previously, we analysed self-organisation of MSC:EC spheroids at a fixed 50:50 cell ratio [19]. To determine the influence of cell concentrations on self-organising behaviour, five different ratios of MSC:EC were co-cultured to form spheroids (20:80, 35:65, 50:50, 65:35 and 80:20 for MSC:EC respectively). To enable cell tracking, MSCs were fluorescently labelled green, the ECs labelled red and the various MSC:EC spheroids were cultured for up to 7 days (Figure 1a). All MSC:EC combinations were able to form spheroids within 24 h, which reduced in size over the time course (Figure S1a,b). However, MSC:EC spheroids at ratios of 20:80 and 35:65 were loosely aggregated and difficult to manipulate, while ECs in lower proportions (35% and 20%), tended to accumulate on the spheroid periphery (Figure S1c). Therefore time-lapse microscopy was used to observe spheroid initiation over the first 20 h in culture using MSC:ECs at a 50:50 ratio. By brightfield imaging, MSC:EC co-cultures aggregated rapidly and condensed into 3D spheroids (Movie S1). Using time-lapse fluorescent microscopy and capturing images every 20 min we found that ECs started to aggregate into discrete foci within 4 h (Figure 1b, key time-points shown). Over time, these foci extended to form interconnected cell clusters. To act as a control for MSC-EC self-organisation, MSC-only spheroids were generated with 50% of the MSCs labelled green and 50% labelled red. These MSC:MSC (50:50) spheroids formed over the same timescale as the MSC-EC spheroids, but in contrast, the red and green labelled cells appeared uniformly distributed at all time points and aggregates of red-labelled cells were not observed (Figure 1b, lower panel). EC networks in MSC:EC spheroids matured over the next 48 h, with increased green-red cell partitioning, as measured by plot-profiling, compared to MSC-only controls (Figure 1c). 

**Figure 2:**
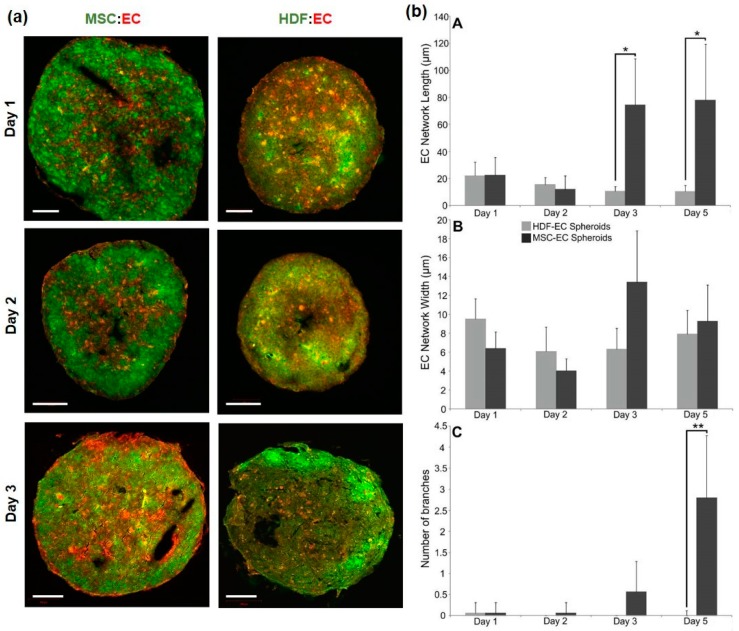
Self-organisation in fluorescently labelled HDF-EC and MSC-EC spheroids. (**a**) Sections of MSC:EC and HDF:EC spheroids grown over 3 days in culture; MSCs or HDFs were labelled green and ECs were labelled red. Scale bars = 100 μm (**b**) Quantification of EC network length (A, top), width (B, middle) and branching (C, bottom) using image analysis.

To determine if ECs self-assembled in a different (non-MSC) stromal environment, we mixed ECs (red) with human dermal fibroblasts (HDFs, green), in place of MSCs, at a 50:50 ratio. We found that EC organisation was induced only in MSC:EC spheroids and not in HDF:EC spheroids (Figure 2a). Quantification by image analysis of the EC network structure demonstrated that network length and branching was significantly increased in MSC:EC spheroids compared to HDF:EC spheroids (Figure 2b). Therefore, EC self-organisation in an MSC environment is dependent on relative cell abundance and EC network formation is optimal at a ratio of 50:50, MSC:EC, which was then used for further analysis.

### 3.2. Analysis of Endogenous Signalling Pathways Controlling Endothelial Cell Organisation in MSC Spheroids

**Figure 3:**
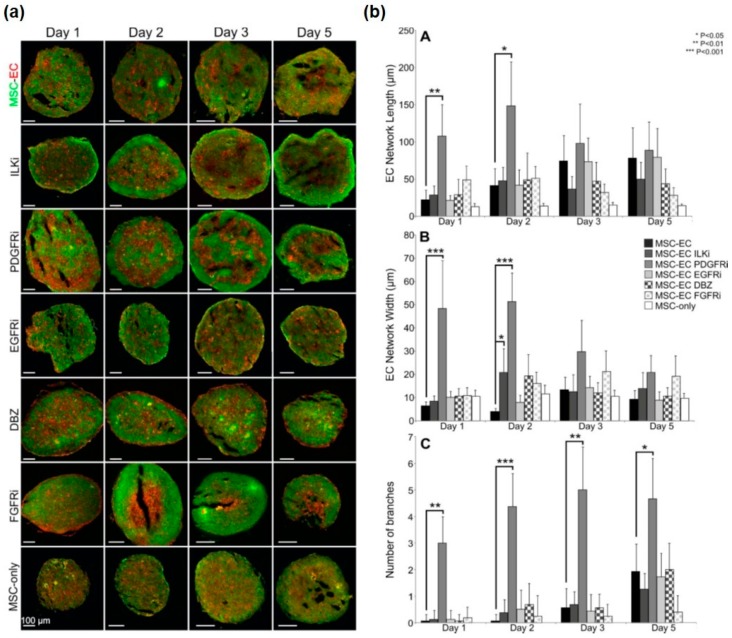
Effect of signalling pathway inhibitors on EC organisation in MSC:EC spheroids. (**a**) Representative sections of spheroids treated with inhibitors over 5 days in culture; MSCs are labelled green, ECs labelled red. Inhibitors targeted ILK, PDGFR, EGFR, Notch (DBZ) and FGFR signalling. MSC-only controls comprised of 50% green and 50% red MSCs. (**b**) Quantification of EC network length (A, top), width (B, middle) and branching (C, bottom) using image analysis.

MSC-EC spheroids were treated with a variety of different inhibitors to block key signalling pathways that have previously been implicated in EC migration and behaviour. These included inhibitors against integrin-linked kinase (ILKi), platelet derived growth factor receptor (PDGFRi), epidermal growth factor receptor (FGFRi), Notch (DBZ) and fibroblast growth factor receptor (FGFRi). Qualitative differences in green-red surface distribution can clearly be observed in whole spheroid images with Volocity rendering (Figure S2). Spheroids were sectioned at different time-points to determine internal organisation patterns. Both ILKi and PDGFRi treatment resulted in the formation of visually more prominent EC networks (Figure 3a). Following image analysis, inhibition of PDGF signalling caused significant increases in the length and width of EC networks at days 1 and 2 and a significantly greater number of branches at all time-points compared to controls; ILKi significantly increased network widths on day 2 only (Figure 3b). Inhibition of Notch signalling with DBZ appeared to promote layering of ECs towards the periphery of the spheroid, whilst FGFRi treatment disrupted EC network formation and induced an aggregation of ECs within the centre of the spheroid. EGFRi treatment appeared to have no effect on EC organisation compared to controls (Figure 3a,b). Collectively, these data demonstrate that MSC-mediated EC organisation is influenced by endogenous signalling activity with PDGF signalling playing a critical role.

### 3.3. Development of Osteo-Chondral-EC Spheroid Models

**Figure 4:**
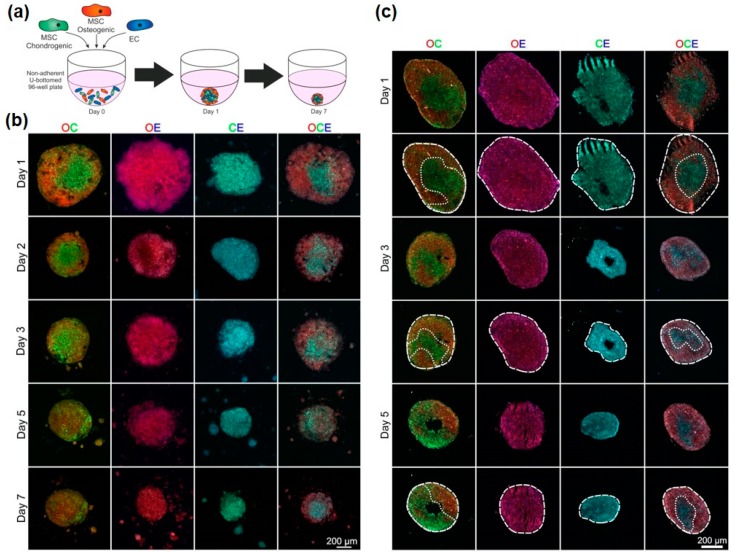
Organisation of ECs in pre-differentiated MSC spheroids. (**a**) Schematic showing technique for combining chondrogenic MSCs, osteogenic MSCs and ECs. MSCs were pre-differentiated for 7 days before being combined with ECs to form various mixed-cell spheroids. Osteogenic MSCs (O) were labelled red, chondrogenic MSCs (C) were labelled green and ECs (E) were labelled blue. (**b**) Images of whole spheroids in OC, OE, CE and OCE combinations. (**c**) Sections of OC, OE, CE and OCE spheroids with dotted line overlays added to distinguish colour separations.

Considering the importance of angiogenesis in endochondral ossification and osteochondral repair, we next determined if the differentiated progeny of MSCs—osteoblasts and chondrocytes—were also able to modulate EC organisation. MSCs were cultured in either osteogenic or chondrogenic induction medium for 7 days, which was sufficient to induce early differentiation, based on alizarin red and alcian blue staining respectively (Figure S3). These pre-differentiated MSCs were combined with ECs to examine the effect of different cell combinations on self-organisation. CellTracker™ red, green and blue were used to identify osteogenic MSCs, chondrogenic MSCs and ECs respectively throughout these experiments (Figure 4a). Various combinations of the three different cell types were combined to create a variety of different 3D mixed-cell spheroids, these were osteogenic and chondrogenic MSCs (OC), osteogenic MSCs and ECs (OE), chondrogenic MSCs and ECs (CE) and all three (OCE) (Figure 4b). All four combinations were found to successfully aggregated into 3D spheroids that were cultured for up to 7 days. Spheroid sections showed that OC and OCE spheroids had clear self-organisation patterns (Figure 4b,c). OC spheroids had lateral-like separation of the osteogenic and chondrogenic MSCs, OCE spheroids appeared to have a predominantly green fluorescent (chondral) core region with a prominent osteogenic periphery. We did not identify ant clear evidence of self-organisation patterns OE and CE spheroids.

**Figure 5:**
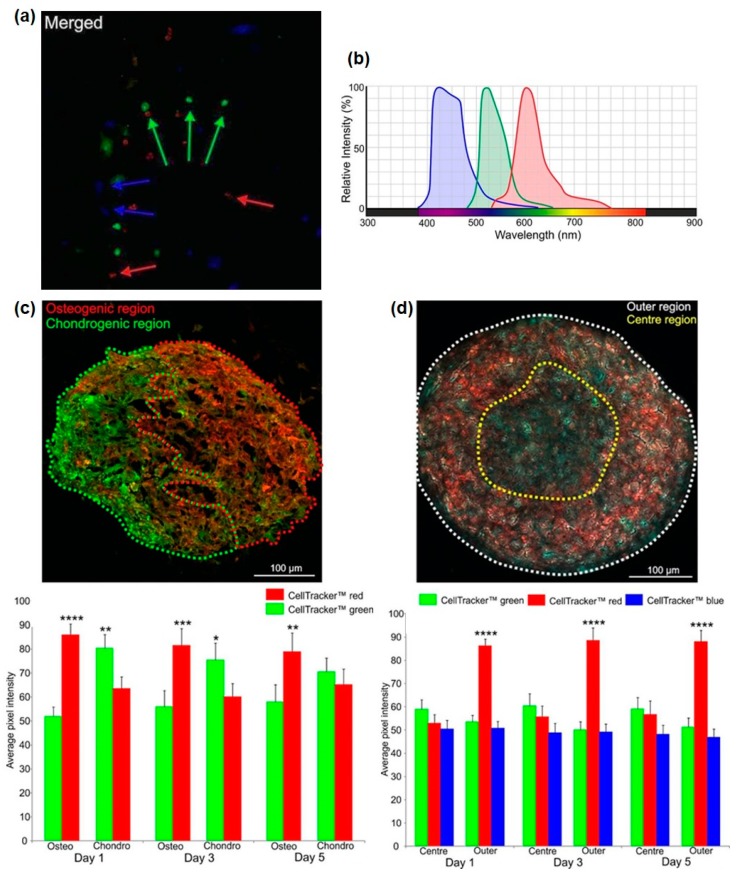
Analysis of OC and OCE spheroid self-organisation. (**a**) CellTracker™ red, green and blue cell labelling in 2D tri-culture to identify the different cell types: osteogenic MSCs (red), chondrogenic MSCs (green) and ECs (blue), arrows indicate selected red, green and blue cells. (**b**) Spectral analysis of the three different CellTracker™ colours showing peak emission wavelengths. (**c**) Day 1 OC section separated into either osteogenic or chondrogenic regions using red or green dotted line. Graph below shows the analysis of spheroid sections at Days 1, 3 and 5 quantifying presence of CellTracker™ red or green within either the osteogenic or chondrogenic regions. (**d**) Day 1 OCE spheroid section separated into either a core region (yellow dotted line) or outer region (white dotted line) based on colour distribution. Graph below shows the analysis of spheroid sections at Days 1, 3 and 5 quantifying presence of CellTracker™ red, green or blue within either the centre or outer regions.

Considering the increased complexity of these cellular organisations, we tested the capacity to spectrally separate different colour combinations of labelled cells to enable quantification of cellular distribution by image analysis, first using simplified, non-confluent 2D co-cultures. The three different regions of the non-descan detector ranging between 400–800 nm were able to individually identify the three different cell types; osteogenic MSCs (red), chondrogenic MSCs (green) and ECs (blue) (Figure 5a). Spectral analysis of the three different CellTracker™ colours confirmed separate peak emission wavelengths with little emission spectrum overlap (Figure 5b). Figure 5c (top) shows an example OC spheroid section at day 1 of culture that has been divided into osteo- and chondrogenic regions based on red-green colour separation. These regions were then analysed over 5 days to quantify the amount of osteogenic MSCs (red) and chondrogenic MSCs (green) (Figure 5c, bottom). These analyses confirmed that there was a significant difference in the partitioning of red and green cells into osteogenic and chondrogenic regions. OCE spheroid sections appeared to show a self-organisation pattern of a central core, possibly consisting of a mixed cell population based on colour, and an outer layer of predominantly osteogenic MSCs (Figure 5d, top). Image analysis was performed on OCE spheroid sections to determine the quantity of CellTracker™ red, green and blue within the two clearly defined regions at days 1, 3 and 5 of culture. For all time points, the outer region had a significantly greater quantity of red fluorescence (osteogenic cells). No significant difference in CellTracker™ green or blue (chondrogenic cells and ECs) distribution was identified between the two regions (Figure 5d, bottom).

## 4. Discussion

In this study we developed models to investigate the interactions between MSCs and ECs in 3D culture and in osteo/chondrogenic differentiation states. Our aim was to determine how intrinsic signalling activity contributes to tissue organisation in controllable in vitro systems that have broad similarities to in vivo biological phenomena such vascular development, endochondral ossification and fracture repair. In our previous studies [19], we identified growth conditions that could maintain MSC and EC viability in 3D spheroid culture using a fixed 50:50 cell ratio. Here, we found that the relative abundance of MSCs and ECs was a critical determinant of EC organising behaviour, which could be due to a reduced number of cell–cell adhesion molecules on ECs. Co-culture of ECs with MSCs has previously been shown to reduce EC permeability through increased VE-cadherin and β-catenin expression [22]. These cell–cell adhesion molecules are essential for cell spheroid formation and subsequent maintenance [23]. Self-organisation is also likely to arise according to the differential adhesion hypothesis, whereby heterogeneous populations of cells segregate into homogeneous groups (in a permissive environment, here 3D co-culture) based on preferential adhesion to isotypic cells [24]. This theory has been tested by altering the number of cadherin cell–cell adhesion molecules on cells within aggregates. Cells with lower cadherin expression enveloped those with higher cadherin expression [25,26]. We did not observe clear cell segregations such as these in our mixed 3D MSC:ECs, instead the ECs first formed internal foci then connected networks throughout the MSC spheroid; stochastic events related to cell migratory tracks and volume filling are also likely to influence cell organisation in a 3D space [27]. It was notable that related stromal cells types, HDFs, were not able to induce and/or support EC self-organisation and this property may be specific to MSCs. 

To determine the role of endogenous signalling activity in directing 3D cell assemblies, a panel of specific inhibitors targeting different signalling pathways were added to MSC-EC spheroids and effects on EC organisation determined. The signalling inhibitors were selected based on known potential to affect pathways linked to angiogenesis and/or migration. A limitation of this approach however is that both MSCs and ECs were exposed to the inhibitors, therefore it is not possible to determine if they were acting on ECs, MSCs or both. Inhibitor concentrations were based on previously published reports, but dose-response effects would also have been informative. Nevertheless, the most significant effects were caused by blockade of endogenous PDGF signalling, inducing increased EC network length, width and branching. These observations may seem counterintuitive as PDGF signalling can control chemotaxis [28] blood vessel morphology [29,30] and plays an important part in stabilising the vasculature [31]. However, loss of or reduced PDGF signalling could result in uncoordinated cell migration and EC organization, as observed in our 3D model. 

Bone is a highly vascularised tissue and ECs play a key role in bone health, disease and repair following injury [32,33]. In these situations, ECs will come into contact with other cellular elements including osteoblast and chondrocyte progenitor cells, which differentiate from MSCs. Recent evidence suggests that ECs promote osteogenesis [34] and coordinate the trans-differentiation of chondrocytes into osteoblasts during fracture repair in mice [35]. To test the consequence of these heterotypic cellular interactions on self-organisation, spheroids combining MSCs pre-differentiated into osteogenic and chondrogenic cells were generated, with and without ECs (OC, OE, CE and OCE). These combinations were able to successfully produce 3D spheroids, however observations of the internal structures demonstrated that only the OC and OCE spheroids underwent cellular self-organisation. In this phase of work, based on the MSC:EC optimization studies, we used equal cell ratios of 50:50 for OC, OE and CE spheroids. However, EC-networks, which were prominent in 3D MSC:ECs were not observed in OE and CE spheroids. It may be that relative cell abundance influences EC self-organization to a different degree when in contact with differentiated MSCs and that further optimization is needed. In addition, there is evidence that MSCs, once differentiated into osteogenic and chondrogenic lineages, have anti-angiogenic effects [36] and here we find that the differentiated status of MSCs, even after only 7 days of pre-differentiation, limits their ability to coordinate EC organisation. We identified a lateral separation of the osteogenic and chondrogenic MSCs in OC spheroids, almost forming a primitive osteochondral junction through intrinsic means alone. In contrast, we found that OCE spheroids had a central mixed cell region with an outer layer primarily composed of osteogenic cells, with no clear demarcation of EC groups. So in these models, the presence of ECs can influence the distribution of osteogenic cells relative to chondrogenic cells. 

Tissue patterning and self-organisation has intrigued scientists for many decades. Alan Turing first theorised in 1952 how morphogen gradients may instruct morphogenesis [37]. Many years later, evidence that a self-organising Turing network could drive pattern formation in mesenchymal progenitors during limb bud development was presented [38]. It is striking that self-organising behaviour is retained in isolated human MSCs, when aggregated in 3D spheroids. These observations can instruct future research using 3D co-culture models and MSC treatment options, particularly those related to mixed cell-based therapies and vasculogenesis.

## Supplementary Materials

The following are available online at http://www.mdpi.com/2306-5354/5/4/92/s1, Figure S1: Development of an optimal 3D co-culture model using MSCs and ECs at different cell ratios; Figure S2: Whole MSC-EC spheroid images rendered using Volocity software. Figure S3: Early osteogenic and chondrogenic differentiation of MSCs prior to incorporation into spheroids. Table S1: Information on inhibitors used in MSC-EC spheroid experiments; Movie S1: Time-lapse brightfield microscopy of MSC:EC (50:50) spheroid formation over the first 17 h of culture, images taken every 15 min.
