# Supplementary material for: Analysis of the Intrinsic Self-Organising Properties of Mesenchymal Stromal Cells in Three-Dimensional Co-Culture Models with Endothelial Cells
Source: Bioengineering (Basel). 2018 Oct 26;5(4):92. doi: 10.3390/bioengineering5040092 (PMC6315484; doi:10.3390/bioengineering5040092)
Supplement: Supplementary file 1 [file bioengineering-05-00092-s001.zip › Suppl Material/Supplementary Materials resubmission PG.docx]

**Supplementary Materials**

**Table S1.** Information on inhibitors used in MSC-EC spheroid experiments

| **Inhibitor** | **Full name** | **Function** | **Justification** |  |
| --- | --- | --- | --- | --- |
|  |  |  |  |  |
| ILKi | N-methyl-3-(1-(4-(piperazin-1-yl)phenyl)-5-(4'-(trifluoromethyl)biphenyl-4-yl)-1H-pyrazol-3-yl)propanamide | Suppresses ILK-mediated phosphorylation of Akt at Ser473 site | Role in ILK in control of vascularisation and MSC function [1, 2]. |  |
| PDGFRi | 3-Fluoro-N-(6,7-dimethoxy-2,4-dihydroindeno[1,2-c]pyrazol-3-yl)phenylamine | Reversible ATP-competitive inhibitor of PDGFR-α and β | Involvement of PDGF signalling in angiogenesis and vascular biology [3-5]. |  |
| EGFRi | 4-[(3-Bromophenyl)amino]-6,7-dimethoxyquinazoline hydrochloride | Potent inhibitor of EGF receptor tyrosine kinase | Role of EGFR in endothelial cell migration and proliferation [6-9]. |  |
| FGFRi | *N*-[2-[[4-(Diethylamino)butyl]amino]-6-(3,5-dimethoxyphenyl)pyrido [2,3-*d*]pyrimidin-7-yl]-*N*'-(1,1-dimethylethyl)urea | Selective FGFR-1 and FGFR-3 inhibitor | Role of FGF signalling in endothelial cells proliferation and migration [10-13]. |  |
| DBZ | *N*-[(1*S*)-2-[[(7*S*)-6,7-Dihydro-5-methyl-6-oxo-5*H*-dibenz[*b*,*d*]azepin-7-yl]amino]-1-methyl-2-oxoethyl]-3,5-difluorobenzeneacetamide | Inhibitor of Notch γ-secretase, preventing cleavage of the Notch ICD | Notch signalling involvement in endothelial cell survival and angiogenesis [14-16]. |  |

**References for Table S1**

1. Yang, X.-M.; Duan, C.-G.; Zhang, J.; Qu, X.-J.; Wang, Y.-S. Integrin-Linked Kinase Controls Choroidal Neovascularization by Recruitment of Endothelial Progenitor Cells. Invest. Ophthalmol. Vis. Sci. 2018, 59, 1779–1789.

2. Zeng, B.; Liu, L.; Wang, S.; Dai, Z. ILK regulates MSCs survival and angiogenesis partially through AKT and mTOR signaling pathways. Acta Histochem. 2017, 119, 400–406.

3. Dong, A.; Seidel, C.; Snell, D.; Ekawardhani, S.; Ahlskog, J. K. J.; Baumann, M.; Shen, J.; Iwase, T.; Tian, J.; Stevens, R.; Hackett, S. F.; Stumpp, M. T.; Campochiaro, P. A. Antagonism of PDGF-BB suppresses subretinal neovascularization and enhances the effects of blocking VEGF-A. Angiogenesis 2014, 17, 553–562.

4. Robinson, S. P.; Ludwig, C.; Paulsson, J.; Ostman, A. The effects of tumor-derived platelet-derived growth factor on vascular morphology and function in vivo revealed by susceptibility MRI. Int. J. Cancer 2008, 122, 1548–1556.

5. Roskoski, R., Jr Sunitinib: a VEGF and PDGF receptor protein kinase and angiogenesis inhibitor. Biochem. Biophys. Res. Commun. 2007, 356, 323–328.

6. Maretzky, T.; Evers, A.; Zhou, W.; Swendeman, S. L.; Wong, P.-M.; Rafii, S.; Reiss, K.; Blobel, C. P. Migration of growth factor-stimulated epithelial and endothelial cells depends on EGFR transactivation by ADAM17. Nat. Commun. 2011, 2, 229.

7. Amin, D. N.; Hida, K.; Bielenberg, D. R.; Klagsbrun, M. Tumor endothelial cells express epidermal growth factor receptor (EGFR) but not ErbB3 and are responsive to EGF and to EGFR kinase inhibitors. Cancer Res. 2006, 66, 2173–2180.

8. Bertrand-Duchesne, M.-P.; Grenier, D.; Gagnon, G. Epidermal growth factor released from platelet-rich plasma promotes endothelial cell proliferation in vitro. J. Periodontal Res. 2010, 45, 87–93.

9. Mehta, V. B.; Besner, G. E. HB-EGF promotes angiogenesis in endothelial cells via PI3-kinase and MAPK signaling pathways. Growth Factors 2007, 25, 253–263.

10. Shing, Y.; Folkman, J.; Sullivan, R.; Butterfield, C.; Murray, J.; Klagsbrun, M. Heparin affinity: purification of a tumor-derived capillary endothelial cell growth factor. Science 1984, 223, 1296–1299.

11. Maciag, T.; Mehlman, T.; Friesel, R.; Schreiber, A. B. Heparin binds endothelial cell growth factor, the principal endothelial cell mitogen in bovine brain. Science 1984, 225, 932–935.

12. Dos Santos, C.; Blanc, C.; Elahouel, R.; Prescott, M.; Carpentier, G.; Ori, A.; Courty, J.; Hamma-Kourbali, Y.; Fernig, D. G.; Delbé, J. Proliferation and migration activities of fibroblast growth factor-2 in endothelial cells are modulated by its direct interaction with heparin affin regulatory peptide. Biochimie 2014, 107 Pt B, 350–357.

13. Giacomini, A.; Chiodelli, P.; Matarazzo, S.; Rusnati, M.; Presta, M.; Ronca, R. Blocking the FGF/FGFR system as a “two-compartment” antiangiogenic/antitumor approach in cancer therapy. Pharmacol. Res. 2016, 107, 172–185.

14. Kerr, G.; Sheldon, H.; Chaikuad, A.; Alfano, I.; von Delft, F.; Bullock, A. N.; Harris, A. L. A small molecule targeting ALK1 prevents Notch cooperativity and inhibits functional angiogenesis. Angiogenesis 2015, 18, 209–217.

15. Chang, L.; Wong, F.; Niessen, K.; Karsan, A. Notch activation promotes endothelial survival through a PI3K-Slug axis. Microvasc. Res. 2013, 89, 80–85.

16. Liu, Z.; Fan, F.; Wang, A.; Zheng, S.; Lu, Y. Dll4-Notch signaling in regulation of tumor angiogenesis. J. Cancer Res. Clin. Oncol. 2014, 140, 525–536.

**
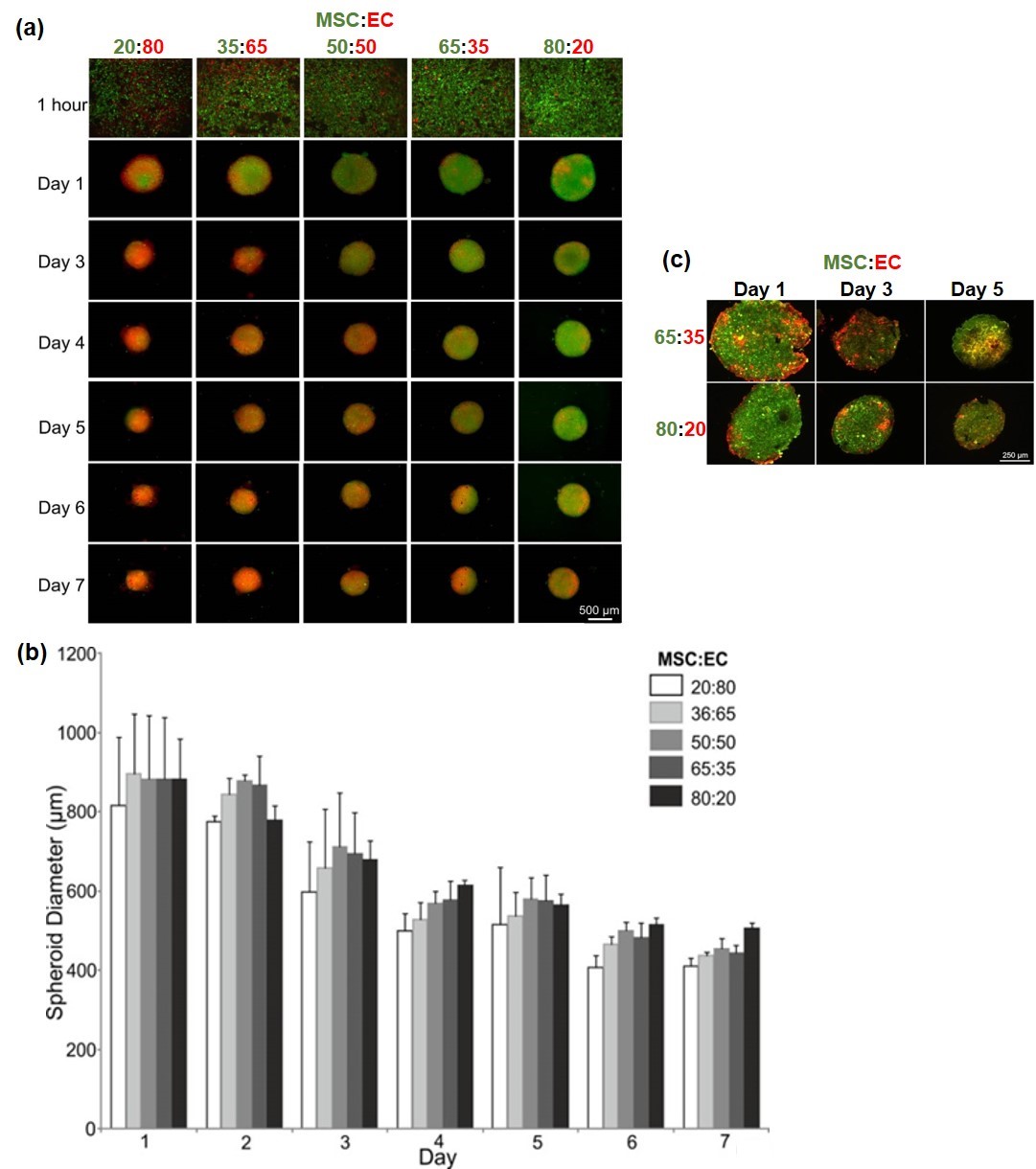
**

**Figure S1. Development of an optimal 3D co-culture model using MSCs and ECs at different cell ratios.** (a) MSC:EC spheroids created by suspending 30,000 cells at MSC:EC ratios of 20:80, 35:65, 50:50, 65:35 and 80:20 in non-adherent U-bottomed plates and cultured for up to 7 days. Whole spheroid images, MSCs labelled green and ECs labelled red. (b) Spheroid diameter measurements over 7 days in culture at different MSC:EC ratios. (c) Sections of the MSC:EC spheroids at 65:35 and 80:30, MSC: EC cell ratios, MSCs labelled green and ECs labelled red.


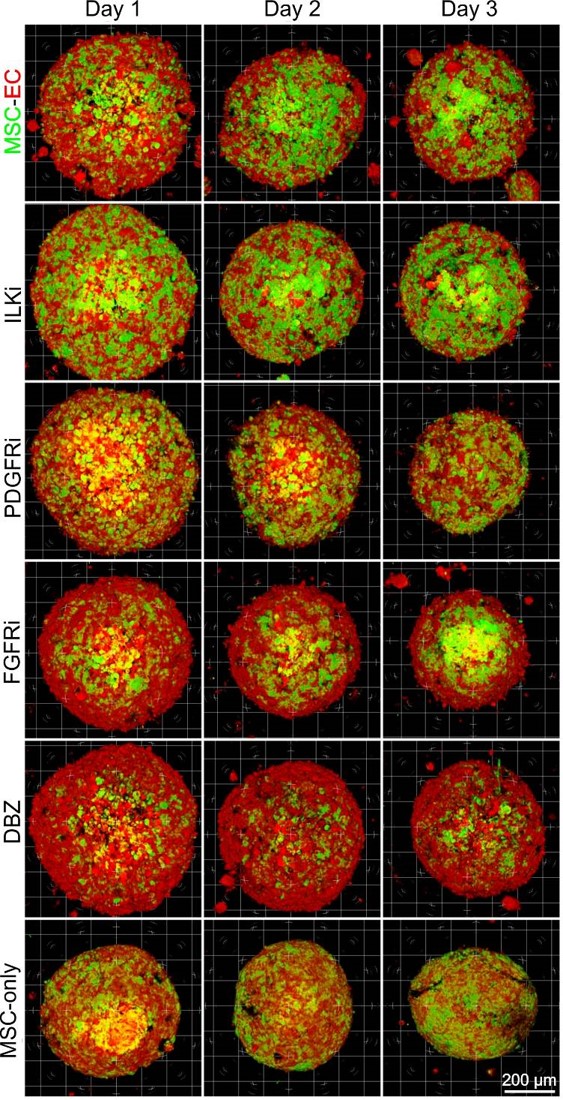


**Figure S2.** Effect of signalling pathway inhibitors on EC surface distribution in MSC:EC spheroids. Whole MSC-EC spheroids treated with inhibitors of different signalling pathways, images were rendered using Volocity image analysis software to show surface cell distributions. MSCs are labelled green, ECs labelled red.


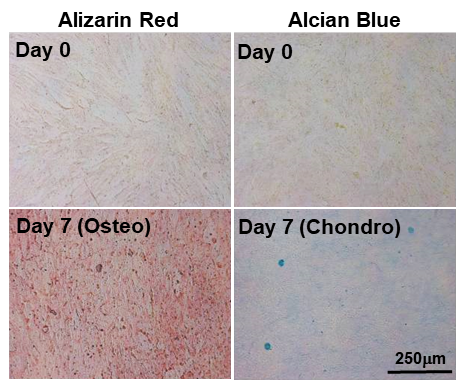


**Figure S3.** Early osteogenic and chondrogenic differentiation of MSCs prior to incorporation into spheroids. MSCs were cultured for 7 days in osteogenic (Osteo) or chondrogenic (Chondro) differentiation medium. Evidence of osteogenesis was determined by Alizarin red staining (red) and chondrogenesis by Alcian blue staining (blue).

**Movie S1.** Time-lapse brightfield microscopy of MSC:EC (50:50) spheroid formation over the first 17 hours of culture, images taken every 15 minutes.

(File uploaded separately)
